# UCN Production With a Single Crystal of Ortho-Deuterium

**DOI:** 10.6028/jres.110.038

**Published:** 2005-06-01

**Authors:** M. Utsuro, M. Tanaka, K. Mishima, Y. Nagai, T. Shima, Y. Fukuda, T. Kohmoto, T. Momose, A. Moriai, K. Okumura, H. Yoshino

**Affiliations:** Research Center for Nuclear Physics, Osaka University, Japan; Research Center for Nuclear Physics, Osaka University, Japan; Department of Sanitary Technology, Kobe Tokiwa College, Japan; Graduate Course of Science, Osaka University, Japan; Research Center for Nuclear Physics, Osaka University, Japan; Department of Physics, Faculaty of Science, Kobe University, Japan; Graduate Course of Science, Kyoto University, Japan; Division of Research Reactor, Japan Atomic Energy Research Institute, Japan; Research Reactor Institute, Kyoto University, Japan

**Keywords:** Bragg scattering, catalyser, cold neutron beam, cryogenic converter, ortho-deuterium, ortho-para conversion, Raman spectroscopy, single crystal, time-of-flight, ultracold neutron guide, ultracold neutrons

## Abstract

The present paper reports on the preliminary experimental results concerning a new concept of ultracold neutron production with a single crystal converter of ortho-deuterium lying in the ground rotational state at the low temperature of about 10 K, which should make it possible to utilize a guided cold neutron beam instead of irradiating the converter material in the inside of high radiation fields. The successful observation of the clear Bragg scattering pattern from the single crystal converter and the reasonable results from the first experimental trial of the ultracold neutron production with the single crystal are shown.

## 1. Introduction

Ultracold neutrons (UCN) with the velocity of about 6 m/s can be stored in a closed vessel called a neutron bottle and therefore are utilized in various kinds of fundamental physics experiments on neutrons [1]. Experimental efforts to develop intense UCN sources with solid deuterium (SD2) as a UCN converter material are carried out worldwide: the Los Alamos National Laboratory (LANL) project for the beta decay asymmetry study at the pulsed spallation source where they recently recorded the highest UCN density [2], the PSI project to utilize the cyclotron spallation neutrons [3,4], and also the reactor SD2 experiment [5] and UCN source projects [6,7]. However, all of these projects and studies are supposed as the SD2 converter material to be inserted into high radiation fields and irradiated with cold neutrons around from directly coupled pre-moderators, which will bring severe thermal and material conditions to the UCN converter.

Ortho-deuterium molecules have much attractive properties as the UCN converter material [8] with a very small absoption cross section for neutrons and lying in the rotational ground state at temperatures below about 20 K. The translational molecular motions also play the important role with the phonon spectrum in SD2 for UCN cooling down, as a superthermal converter providing the UCN density beyond that corresponding to the thermal equilibrium. Solid deuterium has the crystal structure of hexagonal closed pack, with much different sound velocities for the *a*- and the *c*-axes with the lattice parameters of about 0.35 nm and 0.58 nm, respectively, and thus the intersections between the phonon curves[9] and the dispersion curve for a neutron indicating the most effective incident neutron energy for UCN production with a single phonon creation process, are strongly dependent on the relative angle between the crystal axes and the direction of the neutron incidence. Thus, we arrive at a new concept of the UCN production with SD2, setting a single crystal converter of ortho-deuterium in the exit beam of a cold neutron guide from a pulsed source, and rotate the crystal axis synchronized to the time-of-flight spectrum of the incident cold neutrons. The present concept makes us being rid of the high irradiation load problems at the low temperature expected in the internal type of UCN conveter.

The present study reports on the results of our preliminary experiments of the orthodeuterium single crystal preparation as the UCN converter and its Bragg scattering observed, and further the first trial of UCN production with the single crystal of orthodeuterium.

## 2. Experimental Procedures and Results

### 2.1 Preparation of High Purity Ortho-Deuterium

**Fig. 1:**
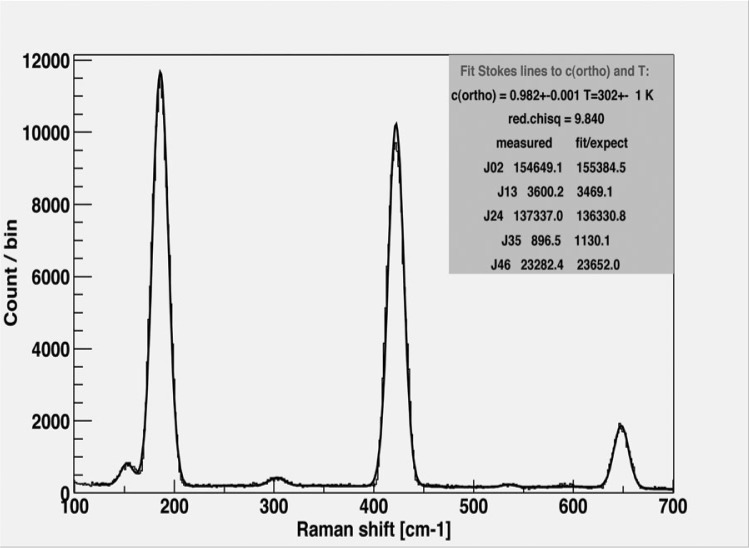
Raman spectrum of the rotational band observed for the catalyser purified orthodeuterium sample.

Since para-deuterium molecules are excited with the energy corresponding to about 90 K even at the lowest rotational state, the effective UCN lifetime in SD2 is determined by the para-content. In the present studies, the high purity ortho-deuterium gas was prepared with the magnetic catalyser OXISORB^2^,^3^ contained in an aluminum cartridge and inserted in the 30 mm *ϕ* × 50 mm*l* copper cell at the top of a two-stage helium refrigerator CryoMini^4^ Model-D510 with the cooling capability down to about 9 K, then ortho-concentration of about 98 % was attained [10] by the direct contact of liquid deuterium with the catalyser at the temperature a little below 20 K. The ortho-concentration was assured by the laser Raman spectroscopy both at the Kobe University physics laboratory in advance to a series of our neutron experiments and also at the JAERI tritium technology laboratory during and after our neutron experiments. One of the typical results of our Raman spectroscopy is shown in Fig. 1, which indicates the ortho-concentration of 98.2 % as the result of fitting analysis.

### 2.2 Preparation of Ortho-Deuterium Single Crystal

The procedure to prepare a several cm cube single crystal of ortho-deuterium was specially developed for the present experiments of the UCN converter at at Prof. Momose’s laboratory in Kyoto University. The key factors for the optimized procedure are the direct and gradual growth of the single crystal from deuterium gas under well controlled optimum gas flow rate and also at the optimum temperature of the crystallising cell. After a number of trials at the Momose laboratory, the obvious perfect quality of the single crystal cube with the optical inspections was at last attained, and the optimim conditions for the crystallising procedure were defined. Then, in our neutron experiments, these conditions are exactly followed for our UCN converter preparation with the 2 cm*^W^* × 6 cm*^H^* × 3 cm*D* crystallising cell connected above our catalyser cell in the refrigerator.

### 2.3 Cold Neutron Bragg Scattering Experiment

**Fig. 2:**
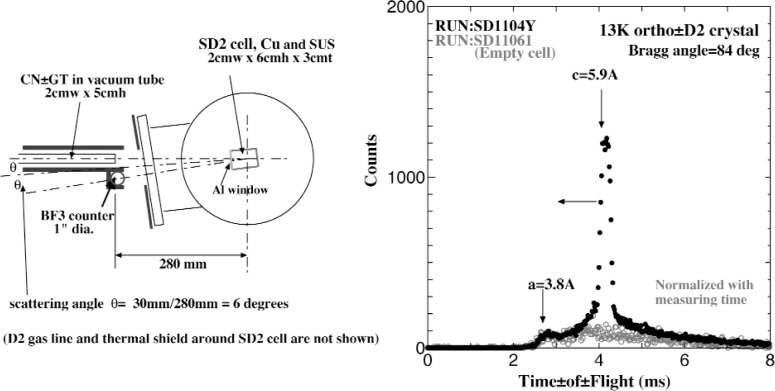
Schematic horizontal arrangement(left) and the measured results(right) of Bragg scattering experiment on ortho-deuterium single crystal UCN converter.

The inspection and the identification of the crystal orientation prepared in our UCN converter cell was carried out with the time-of-flight method to observe the Bragg scattering of the chopper-pulsed cold neutron beam at the exit of C2 guide tube, JRR-3M reactor, JAERI. We specially prepared our experimental setup with ^6^Li tiles and B_4_C plastic collimaters, a narrow slit chopper, a 2 m long evacuated extension guide tube, and related shieldings assembled in the upstream of our cryomachine and also around our gas line shown in Fig. 2 (left).

The typical time-of-flight spectrum measured is shown in Fig. 2 (right) with the total flight path length of about 2.7 m from the chopper to the sample and further to the BF_3_ counter, where the solid circles are the results from the converter cell filled with about 2 cm thick deuterium crystal, while the open circles are those from the empty cell. They indicate clearly the contribution from the very pure lattice structure composed of the *c*-direction of the hexagonal deuterium crystal, just as complete agreement with our expectation to the crystal orientation.

### 2.4 Preliminary Experiments on UCN Production With Cold Neutron Beam

**Fig. 3:**
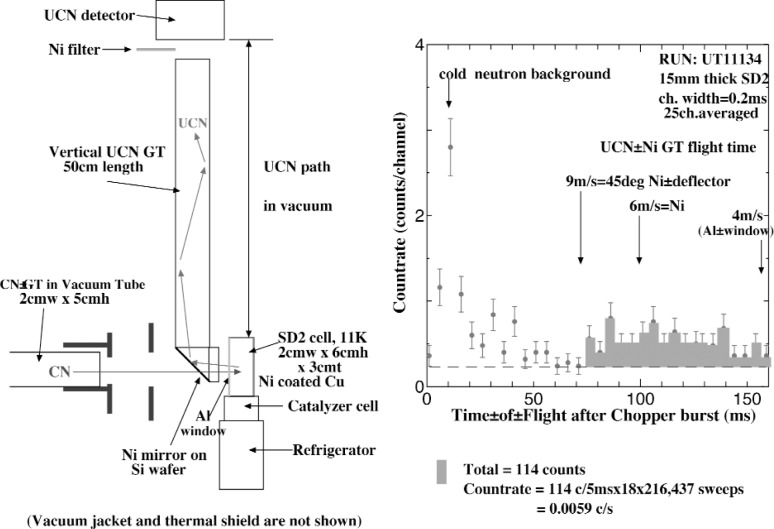
Schematic vertical arrangement(left) and the measured results with the time-of-flight discrimination(right) of UCN measurements produced in ortho-deuterium crystal UCN converter.

Principally, the experiments of UCN production and measurement should be planned with the incident neutron beam as intense as possible since the UCN production rate per incident cold neutron is very small even for the supposed best UCN converter material. However, unfortunately in the present time we must collimate our incident beam very severely to minimize the leakage radiations in order to maintain very low background radiation level in our neutron guide hall to aviod possible influence to neighboring experiments. Actually, our chopper duty factor was about 1/100, and the beam cross section and the divergence reduced to about 1/50 with a series of slits. Our experimental arrangement of the UCN production and measurement after such beam tailoring is shown in Fig. 3 (left), where two kinds of techniques are used for our discrimination of the UCN component, i.e., the time-of-flight discrimination and the filter method reflecting subcritical UCN by a nickel evaporated foil inserted in front of the UCN detector.

Both kinds of the discriminations gave very similar results on the UCN countrate. One of such results obtained on the converter at 11 K for the time-of-flight discrimination is shown in Fig. 3 (right) with the statistical errors, where the countrate in the time region after suffcient attenuation of the scattered cold neutron background and further the constant background subtraction is given as about 0.006/s, being reasonable agreement with our very rough estimation from the original guide exit flux 2 × 10^8^ n/cm^2^s and the reduction factors mentioned above, and further with the velocity factors on the detectable UCN.

## 3. Discussions and Concluding Remarks

Our present experimental situation of the source guide intensity would provide much higher UCN countrate sufficient to show obvious performance of the ortho-deuterium single crystal converter with preparing an improved shielding condition around the extension guide and the converter cell, and recovering the above mentioned reduction factors. Then, measurements on the crystal orientation and incident neutron energy dependences will be much interesting and important tasks for assuring the proposed concept of the ortho-deuterium single crystal UCN converter. Further progress to the continuous beam measurements will give the UCN countrate of about 300/s, enough high to perform a definite demonstration to store the produced UCN.

Another possible utilization of the present concept with the ortho-deuterium single crystal will be the coupling of preferred crystal orientation with the space-dependent cold neutron premoderator spectrum, according to the strong dependence of the most effective incident neutron energy for UCN production on the relative angle between the crystal axes and the inflow direction of cold neutrons.

## Footnotes

2 Certain commercial equipment, instruments, or materials are identified in this paper to foster understanding. Such identification does not imply recommendation or endorsement by the National Institute of Standards and Technology, nor does it imply that the materials or equipment identified are necessarily the best available for the purpose.

3 OXISORB is produced by Messer Griessheim GmbH, Germany. The present specimen was provided by Dr. K. Kirch at PSI.

4 The refrigerator CryoMini Model-D510 is produced by Iwatani Gas Co. Ltd.. The present machine was offered by the Iwatani Gas Company.
